# Detection of Simulated Tactile Gratings by Electro-Static Friction Show a Dependency on Bar Width for Blind and Sighted Observers, and Preliminary Neural Correlates in Sighted Observers

**DOI:** 10.3389/fnins.2020.548030

**Published:** 2020-10-14

**Authors:** Quoc C. Vuong, Aya M. Shaaban, Carla Black, Jess Smith, Mahmoud Nassar, Ahmed Abozied, Patrick Degenaar, Walid Al-Atabany

**Affiliations:** ^1^Biosciences Institute, Newcastle University, Newcastle upon Tyne, United Kingdom; ^2^Biomedical Engineering Department, Faculty of Engineering, Helwan University, Helwan, Egypt; ^3^Newcastle Eye Centre, Royal Victoria Infirmary, Newcastle upon Tyne, United Kingdom; ^4^Faculty of Medicine, Minia University Hospital, Al Minia, Egypt; ^5^Electronics and Communications Department, Faculty of Engineering, Cairo University, Giza, Egypt; ^6^School of Engineering, Newcastle University, Merz Court, Newcastle upon Tyne, United Kingdom

**Keywords:** tactile perception, blind and sighted participants, haptic-feedback technology, fMRI, somatosensory brain network

## Abstract

The three-dimensional micro-structure of physical surfaces produces frictional forces that provide sensory cues about properties of felt surfaces such as roughness. This tactile information activates somatosensory cortices, and frontal and temporal brain regions. Recent advances in haptic-feedback technologies allow the simulation of surface micro-structures via electro-static friction to produce touch sensations on otherwise flat screens. These sensations may benefit those with visual impairment or blindness. The primary aim of the current study was to test blind and sighted participants’ perceptual sensitivity to simulated tactile gratings. A secondary aim was to explore which brain regions were involved in simulated touch to further understand the somatosensory brain network for touch. We used a haptic-feedback touchscreen which simulated tactile gratings using digitally manipulated electro-static friction. In Experiment 1, we compared blind and sighted participants’ ability to detect the gratings by touch alone as a function of their spatial frequency (bar width) and intensity. Both blind and sighted participants showed high sensitivity to detect simulated tactile gratings, and their tactile sensitivity functions showed both linear and quadratic dependency on spatial frequency. In Experiment 2, using functional magnetic resonance imaging, we conducted a preliminary investigation to explore whether brain activation to physical vibrations correlated with blindfolded (but sighted) participants’ performance with simulated tactile gratings outside the scanner. At the neural level, blindfolded (but sighted) participants’ detection performance correlated with brain activation in bi-lateral supplementary motor cortex, left frontal cortex and right occipital cortex. Taken together with previous studies, these results suggest that there are similar perceptual and neural mechanisms for real and simulated touch sensations.

## Introduction

The sense of touch is an integral part of our lives. For those with visual impairments or blindness, touch is also a critical substitute, allowing for important tasks such as reading Braille. When observers interact with objects and surfaces with their hands and fingers, the three-dimensional micro-structure of physical surfaces produces frictional forces that provide sensory cues about properties of the surface such as roughness or hardness (Kaczmarek et al., 1991; Gueorguiev et al., 2017; Culbertson et al., 2018). The perceptual and neural mechanisms for estimating shape and material properties of objects and surfaces from touch sensations have been well studied (see Sathian, 2016, for a review). Recent technological advances have made it possible to simulate this micro-structure on an otherwise flat touchscreen by digitally manipulating electric current or ultrasound to produce frictional forces on the touchscreen (Bau et al., 2010; Meyer et al., 2013; Mullenbach et al., 2013; Vardar et al., 2016, 2017; Vezzoli et al., 2016; see Culbertson et al., 2018, for a review). These forces impede the movement of the finger, giving rise to simulated texture patterns. However, little is known whether blind and sighted observers have similar perceptual mechanisms to interpret these digitally manipulated frictional forces appropriately to perceive the patterns. Furthermore, little is known about possible neural mechanisms and brain regions for processing digitally manipulated frictional forces. Addressing these issues may help develop this haptic-feedback technology to better benefit those with visual impairments. Thus our aim in this study is twofold. First, we compared blind and sighted observers’ tactile sensitivity function to simulated three-dimensional gratings produced by digitally manipulating frictional forces on an otherwise flat touchscreen. Second, in a preliminary functional magnetic resonance imaging (fMRI) study, we used a correlational approach to explore in sighted observers possible brain regions that may be involved in processing simulated tactile gratings.

Much like vision, the somatosensory (tactile) system operates in a hierarchical manner (Carbon and Jakesch, 2013; Breitschaft et al., 2019). That is, as reviewed by Carbon and Jakesch, there is an initial low-level exploration stage which analyze local tactile properties of objects and surfaces (e.g., hardness, roughness, slipperiness, and so on). These properties are elaborated at a mid-level assessment stage which analyses global configurations of the signals (e.g., symmetry, closure or contours). Lastly, there is a high-level evaluation stage which incorporates cognitive and emotional processing of the incoming signals to evaluate the usability and functionality, for instance, of objects and surfaces. This framework has successfully been applied to a range of area where touch is important including aesthetics (Carbon and Jakesch, 2013) and the automotive industry (Breitschaft et al., 2019).

Our study is the first to compare the perceptual tactile system of blind and sighted participants to perceive texture patterns simulated by electro-static friction. Therefore, we focused on the initial low-level exploration stage. Sensory mechanoreceptors (e.g., Meissner or Pacinian corpuscles) embedded in the hand respond to pressure, sheer and vibration on the skin. Like receptors in other modalities, mechanoreceptors are characterized by their temporal resolution (Mountcastle et al., 1972; Brisben et al., 1999) and receptive-field size (Johansson, 1978; Johansson and Valbo, 1979). For example, Pacinian corpuscles have the highest sensitivity to vibrations in the 200–300 Hz temporal frequency range and poorer sensitivity outside this range (Mountcastle et al., 1972; Brisben et al., 1999). In the spatial domain, there is evidence that mechanoreceptors can show either quadratic (“U-shape”) or linear sensitivity functions (Johnson and Phillips, 1981; Connor and Johnson, 1992; Gibson and Craig, 2002). Previous studies using electro-static or ultrasonic frictional forces to simulate touch sensations (Bau et al., 2010; Vardar et al., 2016, 2017) replicated observers’ sensitivity to the temporal frequency of vibrations (Brisben et al., 1999). For example, Vardar et al. (2017) used electro-vibration to simulate sine, saw-tooth and square waveforms at the same intensity with different groove widths between ridges of fixed width (i.e., spatial frequency). Observers rated the roughness of the waveform stimuli as they laterally swept their finger across the stimulus with a velocity of approximately 50 mm/s. They found a quadratic trend for roughness ratings as a function of groove width for all waveforms they used, including the square waveform. Thus, similar perceptual mechanisms may operate for processing both real and simulated touch sensations.

The evidence for tactile acuity differences between blind and sighted observers is mixed. Several studies have shown better performance for grating-orientation discrimination in blind observers (Stevens et al., 1996; Van Boven et al., 2000), which did not depend on the age of blindness onset nor on years of Braille-reading experience (Goldreich and Kanics, 2003, 2006). However, other studies found no differences between blind and sighted observers (Heller, 1989; Grant et al., 2000; Alary et al., 2009). The different findings may relate to the stimuli and tasks used (Alary et al., 2009).

At the neural level, functional magnetic resonance imaging (fMRI) studies show that physical tactile sensations (e.g., vibrations on stationary fingertip) activate a network of brain regions, particularly primary (SI) and secondary (SII) somatosensory cortices (Christova et al., 2013; Chung et al., 2013; Kim et al., 2016; Simoes-Franklin et al., 2011). Other touch-related regions include the insular, cingulate, temporal and parietal regions (Hegner et al., 2010, 2017; Wei and Bao, 2013; Kim et al., 2019). Some of these regions show a dependency on the frequency of vibrotactile stimulation (Harrington and Downs, 2001; Hegner et al., 2007; Soros et al., 2007; Burton et al., 2004, 2008, 2010). The frequency of tactile stimulation can also lead to activation in temporal (auditory) and occipital (visual) cortex (Nordmark et al., 2012). Furthermore, a broad range of measures of neural activity have been shown to correlate with tactile performance outside the scanner. For example, Kim et al. (2015) found that brain activity in supplementary motor cortex can decode the roughness of textured stimuli presented in the scanner, and that decoding accuracy in this region correlated with observers’ performance on an independent roughness discrimination task outside the scanner. Puts et al. (2011) and Heba et al. (2016) showed that concentrations of the inhibitory neurotransmitter GABA in sensorimotor cortex correlated with (temporal) tactile frequency discrimination performance measured outside the scanner. Importantly, this correlation was not found in visual cortex. Lastly, Heba et al. (2016) showed that GABA concentrations in sensorimotor cortex correlated with perceptual improvement following (spatial) tactile 2-point-discrimination training outside the scanner. The haptic-feedback touchscreen we used in the present study is not MR-compatible. Therefore to overcome this limitation for our preliminary fMRI experiment, we focused on correlating brain activation to physical vibrations, as measured by the blood oxygenation-level dependent (BOLD) response, with tactile detection performance, as measured by observers’ tactile sensitivity functions to electro-static induced frictional forces outside the scanner.

Observers in our study actively explored a haptic-feedback touchscreen with their index finger to detect simulated tactile gratings which had different spatial frequencies (bar widths). In Experiment 1, we compared detection performance as a function of spatial frequency (i.e., tactile sensitivity function) between sighted and blind participants. In Experiment 2 using fMRI, we investigated whether brain activation in the somatosensory network to physical vibrations correlated with sighted observers’ tactile sensitivity functions for simulated tactile gratings. Given the literature reviewed, we hypothesized that (1) detection performance would show a dependency on spatial frequency; and (2) that detection performance would be correlated with regions in the network activated by physical touch.

## Materials and Methods

### Experiment 1

#### Participants

Ninety-two adult participants were tested in Egypt and the United Kingdom for Experiment 1. For the Egypt cohort, blind participants were recruited at two Egyptian charities for the visually impaired in Cairo and Al Minya. Sighted participants were recruited from both the general public and universities. Likewise, for the United Kingdom cohort, blind participants were recruited from charities in the North East region of the United Kingdom. For the Blind group, the majority of the participants were between 19 and 55 with five participants older than 55 years old (one each at 58, 60, 64, 68, and 86 years old, and all from the United Kingdom). For the other two visual groups, all of the participants were between 20 and 55 years old. Nine participants were blind from birth. One participant who was 86 years old at the time of testing became blind at 82 years old. The remaining blind participants lost their vision between 4 and 30 years old. As a control for visual cues, we also tested sighted but blindfolded United Kingdom participants (see “control experiment” in the Results). Sighted participants were recruited from the general public or from the institute subject database. None of the sighted participants read Braille. Ethical approval was obtained from the ethical review committees at both Helwan University (Egypt cohort) and Newcastle University (United Kingdom cohort). Table 1 provides demographic information for the different participant groups.

**TABLE 1:** Sex, mean age and mean tactile acuity for the Blind, Sighted and Blindfolded visual groups.

	Egypt/United Kingdom	Congenital blindness (Egypt/United Kingdom)	Braille user (Egypt/United Kingdom)	Female/Male	Age (year)	Tactile Acuity (a.u.)
Blind	26/20	5/4	20/11	24/22	39.8 (14.7)	10.3 (2.8)
Sighted	24/22	–	–	26/20	32.7 (11.2)	11.9 (3.5)
Blindfolded	0/22	–	–	16/6	33.8 (11.4)	10.4 (2.2)

*For the Blind and Sighted visual groups, we combined participants from Egypt and the United Kingdom. For the Blindfolded visual group (control), we only tested United Kingdom participants. The number of congenitally blind participants and Braille users are also indicated for each country. The tactile acuity is the sum of each participant’s responses to Semmes-Weinstein monofilaments, with larger values representing higher acuity (maximum value = 18; see Apparatus). Blind participants were significantly older than sighted participants [t(90) = 2.614, p = 0.010, Cohen’s d = 0.545]. Sighted participants had higher tactile acuity than blind participants [t(90) = 2.411, p = 0.018, Cohen’s d = 0.503]. Values in parentheses are standard deviations.*

#### Apparatus

For the tactile-grating detection task, we used a state-of-the-art surface haptic-feedback tablet (Tanvas, Inc.) to produce tactile sensations. Briefly, this tablet modulates surface friction using electro-static methods (Bau et al., 2010; Meyer et al., 2013; Mullenbach et al., 2013; Vardar et al., 2016, 2017). The frictional forces produced could only be perceived when participants’ moved their fingertip across the touchscreen. The tablet’s haptic-stimulation resolution was designed to match its visual-display and touch-sensing resolution of 2,048 pixels × 1,536 pixels (∼180.2 mm × 135.2 mm). There were 256 levels of intensity of the electro-static friction produced by the tablet at each pixel, with 0 = no electro-static friction and 255 = maximum electro-static friction. Responses were made using a button device connected to the tablet via Bluetooth. We used Semmes-Weinstein monofilaments (Aethesio, Inc.) to assess the (physical) tactile acuity of the participants’ right index finger (Monofilament size: 1.65, 2.36, 2.44, 2.83, 3.61, and 4.56; the larger the size, the easier it is to detect).

#### Stimuli

**FIGURE 1:**
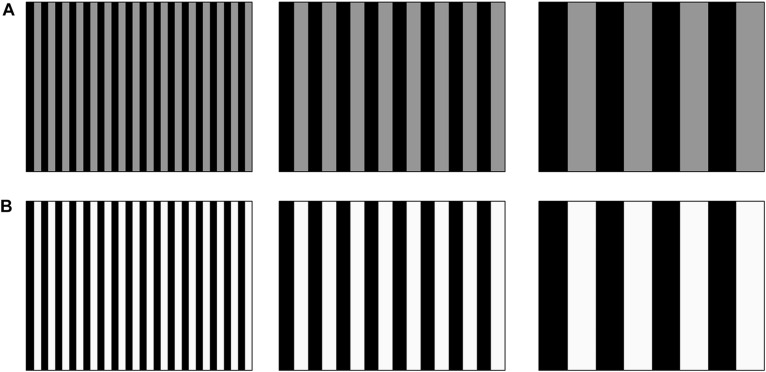
Visual illustration of the tactile gratings. **(A)** Gratings of low tactile-grating intensity (dark gray) with increasing bar width from left to right (decreasing spatial frequency). **(B)** Same as **(A)** with lighter gray bars to represent high tactile-grating intensity. Note that bar width and intensity shown are for illustration purposes only and do not reflect actual values used for the experiment (some of which do not reproduce visually).

Figure 1 provides a visual illustration of the tactile stimuli used in the current study. It should be noted that, visually, the screen remained black on every trial. The stimuli consisted of a tactile grating that covered the entire Tanvas touchscreen in which vertical bars with electro-static friction (represented as grayscale bars in Figures 1A,B) alternated with bars which had no electro-static friction (represented as black bars). We manipulated electro-static friction intensity (from 0 to 255; 0 [no friction], 10 [low], 30 [medium] and 60 [high]) and bar width (0.4, 1.4, 5.6, 11.3 and 45.1 mm). The bar widths corresponded to spatial frequencies of 512, 128, 64, 16, and 4 cycles/image, respectively.

#### Experimental Design and Procedure

Participants were tested individually in a quiet room under normal lighting conditions. The experimenter first asked participants for demographic information such as age, education level and self-reported history of visual impairment. We did not measure visual acuity and thus relied on participants to self-report. Following this, the experimenter tested the participants’ tactile acuity, gave them a break, and then tested them in the tactile detection task.

The experimental procedure for participants in all groups was the same. For the tactile acuity test, participants rested their dominant hand comfortably on a table palm-up. The test began with a practice trial using the Monofilament size 4.56 (which is easily detectable). Participants were asked to close their eyes or look away (if sighted or not blindfolded). The experimenter pressed the monofilament at a 90° angle against the participants’ index finger until it bowed. It was held in place for 1.5 s and then removed. The experimenter then asked (1) if participants felt a transient force on their fingertip and (2) if they felt a sustained force on their fingertip. The experimenter then repeated this step with the same monofilament after a 10 s delay. For all participants, the experimenter tested the monofilaments in the following fixed-size order: 2.36, 2.83, sham, 3.61, 1.65, sham, 2.44. Each monofilament size was tested twice (again with a 10 s delay between any testing). For the sham test, the experimenter asked the participants the two questions but did not apply any monofilament.

For the tactile detection task, the experimenter first cleaned the touchscreen and asked participants to clean their fingertip of the dominant hand using a lens wipe. Participants were allowed to tactually explore the touchscreen so that they were familiar with the size of the screen. The experimenter then explained the stimuli and task to participants and used the tablet’s built-in tactile demonstration software which also displayed grating patterns as a practice to give them experience with the electro-static friction it produced. Participants were encouraged to make lateral sweeping finger movements across the screen at a repeatable speed and were allowed to practice that (e.g., Vardar et al., 2016, 2017). We were able to accurately record the finger’s screen position (*x*- and *y*-coordinates), but due to a coding error we were not able to accurately compute the finger’s velocity. For descriptive purposes, we therefore present positional heat maps as Supplementary Figures (see Supplementary Data for more details).

The experimental trials began once participants were comfortable with the procedure. The tablet was placed on the table in front of them and held in place by the experimenter throughout the experiment. For each trial, participants were asked to verbally indicate whether they felt a tactile grating or not as accurately as possible. There was no time pressure, so participants could freely explore the touchscreen for as long as they like. The experimenter pressed the “yes” or “no” key on the button device after the participants responded to advance to the next trial. There was an enforced 1 min break after every 40 trials. During this period, the experimenter cleaned the touchscreen with a wipe to ensure there was no build-up of fatty deposits which may have reduced the signal sensation. Participants could also ask for a break at any point during the experiment. There were 10 repetitions for each of the 15 tactile gratings (5 bar widths × 3 intensities) and 50 trials when no grating was presented for a total of 200 trials. These trials were presented in a different random order for each participant. For sighted participants, there were no instructions to close their eyes during the tactile detection task. For blindfolded participants (“control experiment”), we placed the blindfold on before bringing out the Tanvas touchscreen (i.e., after measuring physical tactile acuity) so that they were unaware of the physical dimensions of the touchscreen other than from their tactual exploration of the screen. Blindfolding also controlled for potential contributions of a visual reference frame to detection performance when sighted participants performed the task. The whole session lasted approximately 1.5 h.

#### Statistical Analysis

We used signal detection theory to analyze participants’ sensitivity to electro-static friction, using the cumulative d’ measure (Macmillan and Creelman, 2005). The cumulative d’ uses the false alarm rate when no grating was presented for each of the other 15 tactile-grating conditions (3 intensities × 5 bar widths). This measure was computed for each participant as follows. We defined the hit rate as the proportion of trials participants responded “yes” when a tactile grating was presented separately for each condition (10 trials per condition). We then defined one false alarm rate as the proportion of trials participants responded “yes” when no gratings were presented (i.e., intensity = 0). This rate was based on 50 trials. Hit and false alarm rates of 0.0 were then replaced by 1/2N and rates of 1.0 were replaced by 1 – 1/2N (where N = the number of repetitions). Lastly, for each participant, we calculated the cumulative d’ for each of the 15 tactile-grating conditions (5 bar widths x 3 intensities) as:

(1) $$C\,u\,m\,u\,l\,a\,t\,i\,v\,e\,d^{\prime }=Z\,\left(h\,i\,t\,r\,a\,t\,e\right)-Z\,\left(f\,a\,l\,s\,e\,a\,l\,a\,r\,m\,r\,a\,t\,e\right)$$

We pooled the cumulative d’ data across countries and submitted the data to an omnibus 2 visual condition × 5 bar width × 3 intensity mixed analysis of variance (ANOVA) with visual condition (Blind and Sighted) as between-subjects factors, and bar width (45.1, 11.3, 2.8, 1.4, and 0.4 mm) and intensity (low, medium and high) as within-subjects factors. For the United Kingdom cohort, we also conducted ANOVAs with three between-subjects visual conditions (Blind, Sighted and Blindfolded). For bar width and intensity, we also report polynomial trends in the data. We used Greenhouse-Geisser correction. Following the omnibus ANOVAs, we carried out further *post hoc* simple ANOVAs or *t*-tests to help interpret the results. For *post hoc t*-tests, we used Bonferroni correction. An *alpha* = 0.05 was used as our level of significance for all statistical tests. We report $\eta_{\text{p}}^{2}$ (partial-eta squared) as our measure of effect size.

To better quantify any linear and quadratic relationship between cumulative d’ and bar width, we also fitted each of these participant’s cumulative d’ with a second-order polynomial function:

(2) $$y=p_{1}\,x^{2}+p_{2}\,x+p_{3}$$

where *y* represents the cumulative d’ for a given bar width *x*, and the parameters *p*_1_, *p*_2_, and *p*_3_ represent the quadratic, linear and constant term, respectively. We used log_1__0_(*x*) so that bar width increased linearly (rather than exponentially).

### Experiment 2

#### Participants

Nineteen participants were tested in Experiment 2 (age: *M* = 22.7 years old, *SD* = 2.5 years old; 8 females). This group was tested on the tactile detection task while blindfolded, and they were different from the Blindfolded group tested in Experiment 1. The tactile acuity was not measured for these participants. The behavioral and fMRI phases were conducted in separate sessions on different days between 1 and 7 days apart, with the order randomly determined for each participant. The fMRI component was part of a larger study; thus, for some of the fMRI analyses which were not dependent on the behavioral phase (see below), we used the larger sample of participants to increase statistical power.

### Behavioral Phase

The stimuli, apparatus, design and task were the same as in Experiment 1. The exceptions were: (1) there were 15 (rather than 10) repetitions for each of the 15 tactile gratings (5 bar widths × 3 intensities) and 75 trials when no grating was presented for a total of 300 trials; and (2) we did not measure tactile sensitivity using the Semmes-Weinstein monofilaments. Participants were tested blind-folded.

### fMRI Phase

#### Stimuli

During the fMRI phase, we delivered vibrations to participants’ stationary finger with the force indentational to the finger skin. Two types of physical, tactile stimuli were generated during this phase. The first type of tactile stimulus, which we refer to as an envelope stimulus, was created by modulating the amplitude of a carrier vibrotactile signal with the speech envelope from a spoken sentence arbitrarily selected from the IEEE sentence database (Rothauser, 1969). The same sentence was used for all presentation of this tactile stimulus. To extract the envelope, the auditory signal was Hilbert transformed and then full-wave rectified. A second-order low-pass Butterworth filter (with a frequency cut off of 30 Hz) was then applied to the rectified signal. The envelope was then used to modulate the amplitude of a vibrotactile signal which had a carrier frequency of 120 Hz. Thus this stimulus type had power mostly in the low frequencies up to 30 Hz. The second type of tactile stimulus, which we refer to as a sinusoidal stimulus, was created by using a 2 Hz sinusoidal wave to modulate the amplitude of vibrotactile signals which had carrier frequencies of either 60, 120, or 200 Hz (which we denote as F60, F120, and F200, respectively). Thus this stimulus type had power concentrated at 2 Hz for all carrier frequencies. The four tactile stimuli had a duration of 2.39 s.

#### Apparatus

Instructions and other visual elements were back-projected onto a screen at the foot end of the scanner using a Canon XEED LCD projector (1,280 × 1,024 pixels, 60 Hz). The tactile stimuli were delivered to participants’ left index fingertip using a mini-PTS MR-compatible tactile transducer system (Dancer Design). This system had a tactor that was ∼6 mm in diameter. The output of the system’s amplifier was set to a comfortable level based on pilot study feedback (Level 6 on the system). The tactor was attached to a wooden block, making it easier for participants to keep their finger in contact with the tactor by grasping hold of the block. They wore ear plugs to protect against scanner noise. Head motion was restricted by placing foam pads between the head and head coil. The experiment was run on a Windows 7 PC using the Psychophysics Toolbox version 3 (run on 32-bit MATLAB 2012, Mathworks, Inc.; Brainard, 1997; Pelli, 1997; Kleiner et al., 2007) to control the experiment, present the stimuli and record behavioral responses.

#### Experimental Design and Procedure

For each participant, the four types of tactile stimuli (envelope, sine wave 60 Hz, sine wave 120 Hz and sine wave 200 Hz) were each presented three times in a random order for a total of 12 stimulus blocks (14.5 s per block). The stimulus was presented five times in each block with a 500 ms pause between each presentation. A white fixation cross was presented throughout each tactile-stimulation block. Fixation-only blocks (without tactile stimulation) were presented at the beginning of the session and after each stimulus block (13 blocks; 9.0 s per block). Participants were instructed to fixate on the cross and attend to the tactile stimulation but otherwise did not perform any tasks.

#### Image Acquisition

Scanning took place at the Newcastle University Magnetic Resonance Centre. A 3 T Philips Intera Acheiva MR scanner acquired anatomical T1-weighted images and functional T2^∗^-weighted echo planar images (EPIs) using a Philips 8-channel receive-only head coil. The high- resolution T1-weighted scan contained 150 slices with an acquisition time of approximately 5 min. The structural scan parameters were: repetition time (TR) = 9.6 ms, echo time (TE) = 4.6 ms, flip angle = 8°. The field of view (FOV) was 240 mm × 240 mm × 180 mm with a matrix size of 208 × 208 pixels. Each voxel was 0.94 mm × 0.94 mm × 1.2 mm in size. The T2^∗^-weighted EPIs were acquired from the bottom to the top of the head and comprised of 29 axial slices. The parameters of the EPIs were: acquisition time (TA) = 1.3 s, TR = 1.92 s, TE = 40 ms, flip angle = 90°. The FOV had a matrix size of 64 × 64 pixels and was 192 mm × 115 mm × 192 mm. Each voxel was 3 mm × 3 mm × 3 mm in size. There was a 1.0 mm gap between slices. To increase the signal-to-noise ratio of the functional images, sensitivity encoding (SENSE) was used with factor = 2. For each run, a total of 160 functional images were acquired (319 s). Four “dummy” scans were acquired prior to each functional run to allow for equilibration of the T1 signal.

#### fMRI Pre-processing

To correct for head motion for each participant, functional images were realigned by rigid rotation and translation along all three axes to the first image. These realigned images were normalized to a standard Montreal Neurological Institute (MNI) EPI T2^∗^-weighted template, and the voxel size was resampled to 3 mm × 3 mm × 3 mm. Images were then spatially smoothed with a 6 mm full-width-at-half-maximum Gaussian kernel to improve the signal-to-noise ratio. A high-pass filter with a cut off of 180 s was used to remove low-frequency drifts in the signal.

#### fMRI Statistical Analyses

SPM12 was used to analyze the pre-processed data (Friston et al., 1994). A general linear model (GLM) was used in a two-step mixed-effects approach. A fixed-effects model was firstly used to analyze each participant’s data set. The individual datasets were then analyzed at the group level using a random-effects model. No additional smoothing of the images was used at the group level.

For each participant, a design matrix was created which modeled the onset and duration of the four stimulation block types (14.5 s) and the fixation block type (9.0 s) as separate boxcar functions. These functions were convolved with the canonical hemodynamic response to create five regressors of interest. The six movement parameters (roll, yaw, pitch, and three translation terms) from the realignment were also included in the design matrix as regressors of no interest. Lastly, we fitted a linear combination of the regressors to the functional data to estimate the beta weight for each of the five block types and create a corresponding beta image. The resulting value at each voxel in subsequent contrast images reflect the magnitude of brain activation during vibrotactile stimulation by the stimulus at that voxel.

At the group level, we first conducted a region of interest (ROI) analysis to investigate whether BOLD responses in touch-related brain regions correlate with detection performance. We functionally localize touch-related ROIs using data from all fMRI participants (*N* = 25) to increase statistical power. One-sample *t*-tests of contrast images derived from participants’ estimated beta images were conducted at each voxel. For the group-level functional localization, we used an initial threshold of *p* < 0.001, a minimum cluster size of *k* = 20 voxels, and accepted clusters if *p* < 0.05 uncorrected at the cluster level. Subsequent analyses used only the 19 participants who were also tested with the Tanvas tablet outside the scanner. We also fitted participant’s cumulative d’ with a second-order polynomial function (Eq. 2). We extracted the mean beta estimate from each ROI and conducted a correlation between that ROI’s mean activation and each parameter *p*_*i*_.

Second, we conducted a whole-brain multiple regression to localize voxels that may fall outside of any functionally localized ROIs, which are correlated with participants’ detection performance. We used each participant’s fitted parameters (*p*_1_, *p*_2_, and *p*_3_) as regressors in a second-level design matrix to predict their brain activation. These parameters captured participants’ performance better and reduced the number of regressors (three instead of five). One-sample *t*-tests were then used to test whether fitted beta weights for the regressors were significantly greater or less than zero. Positive beta weights represent a positive correlation with performance, whereas negative beta weights represent a negative correlation with performance. For this group-level analysis, we used an initial threshold of *p* < 0.01 and a minimum cluster size of *k* = 20 voxels (unless otherwise stated). We accepted clusters as significant if *p* < 0.05, FDR-corrected for multiple comparisons at the cluster level. For all analyses, we used SPM12’s neuromorphic anatomy function to label brain structure associated with the clusters of interest.

## Results

### Experiment 1

#### Comparisons Between Blind and Sighted Participants

**FIGURE 2:**
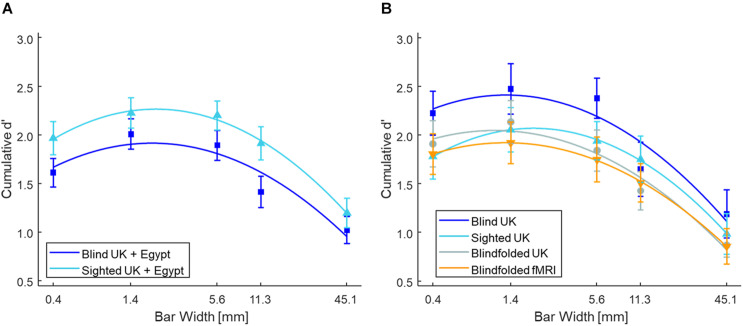
Tactile grating detection performance across Experiments 1 and 2 at low intensity. Participants’ detection performance (cumulative d’) as a function of bar width (mm). Only performance from the low tactile-grating intensity are plotted as there is a strong linear and quadratic trend at this tactile-grating intensity. **(A)** Blind (*N* = 46) and sighted (*N* = 46) participants in Experiment 1 averaged across both Egypt and the United Kingdom. **(B)** The control experiment with sighted but blindfolded (*N* = 22) United Kingdom participants compared to the blind (*N* = 20) and sighted (*N* = 22) United Kingdom participants. The blindfolded participants from the fMRI study of Experiment 2 (*N* = 19) are also plotted for comparison. In all plots, the error bars represent standard error of the mean, and the solid lines represent the fitted second-order polynomial function (Eq. 2). There were no significant differences between blind and sighted participants. See Table 2 for the grating detection performance across both experiments at the medium and high tactile-grating intensity.

Figure 2A shows the mean cumulative d’ as a function of bar width at low tactile-grating intensity for the Blind and Sighted groups (pooled across both countries). Table 2 presents the data for the medium and high tactile-grating intensity. Given that we used cumulative d’ which uses the same false alarm rate for all conditions, Table 3 presents the false alarm and hit rates for the different conditions. There was a marginal effect of visual group with sighted participants performing slightly better than blind participants [*F*(1, 90) = 3.528, *p* = 0.064, $\eta_{\text{p}}^{2}=$0.038; Blind: *M* = 2.56 *SE* = 0.11; Sighted: *M* = 2.83, *SE* = 0.11). This marginal effect may be related to age and tactile-acuity differences between groups, despite our efforts to match them as best as possible (see Table 1). Blind participants were significantly older (*p* = 0.010) and had lower tactile acuity than sighted participants (*p* = 0.018). Importantly for our purposes, visual group did not significantly interact with the two other factors (*F*s < 1.943, *p*s > 0.100). There were main effects of tactile-grating intensity [*F*(1.121, 100.861) = 253.957, *p* < .001, $\eta_{\text{p}}^{2}=$0.738] and bar width [*F*(2.847, 256.262) = 53.572, *p* < .001, $\eta_{\text{p}}^{2}=$ 0.373]. Lastly, there was a significant two-way interaction between intensity and bar width [*F*(4.130, 371.709) = 26.943, *p* < 0.001, $\eta_{\text{p}}^{2}=$ 0.230].

**TABLE 2:** Tactile grating sensitivity across Experiments 1 and 2 at medium and high intensity.

	Medium					High				
	0.4	1.4	5.6	11.3	45.1	.4	1.4	5.6	11.3	45.1
Blind (*N* = 46)	2.94 (0.14)	3.02 (0.14)	2.99 (0.13)	2.98 (0.14)	2.66 (0.16)	3.17 (0.11)	3.19 (0.12)	3.13 (0.13)	3.18 (0.10)	3.00 (0.13)
Sighted (*N* = 46)	3.27 (0.11)	3.27 (0.10)	3.31 (0.10)	3.28 (0.10)	3.14 (0.11)	3.32 (0.10)	3.32 (0.10)	3.33 (0.10)	3.33 (0.10)	3.33 (0.10)
Blindfolded (Exp 1 *N* = 22)	3.13 (0.21)	3.21 (0.19)	3.21 (0.19)	3.16 (0.20)	2.97 (0.21)	3.21 (0.19)	3.21 (0.19)	3.19 (0.20)	3.19 (0.18)	3.19 (0.18)
Blindfolded (Exp 2 *N* = 19)	3.09 (0.16)	3.16 (0.16)	3.15 (0.15)	3.17 (0.15)	3.05 (0.15)	3.15 (0.18)	3.13 (0.17)	3.19 (0.16)	3.16 (0.16)	3.08 (0.17)

*Participants’ detection performance (cumulative d’) as a function of bar width (mm). Performance at the medium tactile-grating intensity showed weak linear and quadratic trends. Performance at the high tactile-grating intensity showed no significant linear or quadratic trends. Note that there were two different Blindfolded groups (one in each experiment).*

**TABLE 3:** Tactile grating detection performance across Experiments 1 and 2.

		Bar widths (mm)					
		No Bars	0.4	1.4	5.6	11.3	45.1
Blind	Low	0.09 (0.02)	0.49 (0.05)	0.61 (0.05)	0.58 (0.05)	0.43 (0.05)	0.30 (0.04)
	Medium		0.90 (0.03)	0.92 (0.03)	0.91 (0.03)	0.91 (0.03)	0.81 (0.04)
	High		0.96 (0.02)	0.97 (0.02)	0.95 (0.02)	0.97 (0.01)	0.91 (0.03)
Sighted	Low	0.08 (0.02)	0.58 (0.05)	0.66 (0.05)	0.65 (0.04)	0.56 (0.05)	0.34 (0.05)
	Medium		0.98 (0.01)	0.98 (0.01)	0.99 (0.01)	0.98 (0.01)	0.94 (0.01)
	High		0.99 (0.01)	0.99 (0.01)	0.99 (0.01)	0.99 (0.01)	0.99 (0.01)
Blindfolded (Exp 1)	Low	0.12 (0.04)	0.61 (0.07)	0.68 (0.07)	0.59 (0.07)	0.46 (0.07)	0.28 (0.06)
	Medium		0.98 (0.01)	10.00 (–)	10.00 (–)	0.99 (0.01)	0.94 (0.03)
	High		10.00 (–)	10.00 (–)	10.00 (–)	10.00 (–)	10.00 (–)
Blindfolded (Exp 2)	Low	0.10 (0.03)	0.59 (0.07)	0.61 (0.07)	0.57 (0.08)	0.48 (0.07)	0.30 (0.07)
	Medium		0.97 (0.01)	0.99 (0.01)	0.99 (0.01)	0.99 (0.01)	0.96 (0.01)
	High		0.99 (0.01)	0.98 (0.01)	10.00 (–)	0.99 (0.01)	0.97 (0.01)

*Participants’ false alarm and hit rates for the different visual groups. The false alarm rate is reported for the “No Bar” condition (i.e., responding “yes” when there is no bar present). The hit rate is reported as a function of intensity and bar width (i.e., responding “yes” when a bar is present). Note that there were two different Blindfolded groups (one in each experiment).*

To determine the shape of participants’ tactile sensitivity function, we used polynomial contrasts to test for linear and quadratic trends in the cumulative d’ data. Not surprisingly, there was a linear and quadratic relationship between cumulative d’ and intensity [linear contrast: *F*(1, 90) = 273.296, *p* < 0.001, $\eta_{\text{p}}^{2}=$ 0.752; quadratic contrast: *F*(1, 90) = 191.758, *p* < 0.001, $\eta_{\text{p}}^{2}=$ 0.681]. Notably, there was also a linear and quadratic relationship between cumulative d’ and bar width [linear contrast: *F*(1, 90) = 62.849, *p* < 0.001, $\eta_{\text{p}}^{2}=$0.411; quadratic contrast: *F*(1, 90) = 116.136, *p* < 0.001, $\eta_{\text{p}}^{2}=$0.563]. Given the interaction between intensity and bar width, we further conducted *post hoc* simple ANOVAs separately for each intensity level with bar width as a within-subjects factor and pooling across visual groups. For low tactile-grating intensity, there were significant linear and quadratic trends [linear contrast: *F*(1, 91) = 81.176, *p* = 0.003, $\eta_{\text{p}}^{2}=$0.471; quadratic contrast: *F*(1, 91) = 96.337, *p* < 0.001, $\eta_{\text{p}}^{2}=$0.514]. Similarly for medium intensity, there were significant linear and quadratic trends but the effect size ($\eta_{\text{p}}^{2}$) was much smaller than the low intensity [linear contrast: *F*(1, 91) = 9.158, *p* = 0.003, $\eta_{\text{p}}^{2}=$0.091; quadratic contrast: *F*(1, 91) = 19.616, *p* < 0.001, $\eta_{\text{p}}^{2}=$ 0.177]. Finally there were no significant linear or quadratic trends for high intensity (*F*s < 2.409, *p*s > 0.124). Overall irrespective of visual group, participants’ performance increased with increasing tactile-grating intensity, and they generally performed best when bar widths were middling sizes. Lastly, the effect of bar width on performance was most pronounced when the task was difficult (i.e., low intensity).

### Control Experiment: Comparisons Between Blind, Sighted, and Sighted but Blindfolded United Kingdom Participants

Sighted participants had a visual reference frame (e.g., seeing the size of the touchscreen). To test whether visual information affected detection performance, we tested an additional sighted but blindfolded group in the United Kingdom (see Table 1) and compared their detection performance with the blind and sighted United Kingdom participants’ performance. Given potential for cultural differences, we did not include the Egypt participants in this analysis. For the Blindfolded group, the experimenter ensured that participants were willing to be blindfolded prior to inviting them to take part in the experiment. If they agreed, they came to the lab, provided written consent and asked to put on a sleeping mask before the tactile sensitivity and tactile detection tasks. The experimenter ensured that they did not see the tablet at any point during the experiment.

Figure 2B shows the mean cumulative d’ for the three United Kingdom visual groups (see also Table 2). The overall pattern of detection performance was similar irrespective of whether sighted participants were blindfolded or not. There was no main effect of visual group, and visual group did not interact with intensity or bar width (all *F*s < 1.147). The main effects intensity and bar width, and their interaction were significant [intensity: *F*(1.020, 62.206) = 148.876, *p* < 0.001, $\eta_{\text{p}}^{2}=$ 0.709; bar width: *F*(2.613, 159.387) = 62.894, *p* < 0.001, $\eta_{\text{p}}^{2}=$0.508; interaction: *F*(10.729, 178.400) = 33.697, *p* < 0.001, $\eta_{\text{p}}^{2}=$ 0.356]. Similar to our main analysis above, there was a linear and quadratic relationship between cumulative d’ and intensity [linear contrast: *F*(1, 61) = 150.717, *p* < 0.001, $\eta_{\text{p}}^{2}=$ 0.712; quadratic contrast: *F*(1, 61) = 142.804, *p* < 0.001, $\eta_{\text{p}}^{2}=$0.701]. There was also a linear and quadratic relationship between cumulative d’ and bar width [linear contrast: *F*(1, 61) = 94.698, *p* < 0.001, $\eta_{\text{p}}^{2}=$0.608; quadratic contrast: *F*(1, 61) = 81.850, *p* < 0.001, $\eta_{\text{p}}^{2}=$0.573].

### Experiment 2

#### Detection Performance Outside the Scanner

Figure 2B shows the mean cumulative d’ for the fMRI group (see also Table 2). The main effects and interaction were significant [intensity: *F*(1.021, 18.384) = 60.741, *p* < 0.001, $\eta_{\text{p}}^{2}=$ 0.771; bar width: *F*(2.933, 52.794) = 22.436, *p* < 0.001, $\eta_{\text{p}}^{2}=$ 0.555; interaction: *F*(3.323, 59.812) = 12.413, *p* < 0.001, $\eta_{\text{p}}^{2}=$ 0.408]. There were linear and quadratic relationships between cumulative d’ and intensity [linear contrast: *F*(1, 18) = 59.854, *p* < 0.001, $\eta_{\text{p}}^{2}=$ 0.769; quadratic contrast: *F*(1, 18) = 63.719, *p* < 0.001, $\eta_{\text{p}}^{2}=$ 0.780], and between cumulative d’ and bar width [linear contrast: *F*(1, 18) = 36.573, *p* < 0.001, $\eta_{\text{p}}^{2}=$ 0.670; quadratic contrast: *F*(1, 18) = 22.403, *p* < 0.001, $\eta_{\text{p}}^{2}=$ 0.554]. Overall the pattern of detection performance was similar to participants in Experiment 1.

#### fMRI Region of Interest Analysis

**FIGURE 3:**
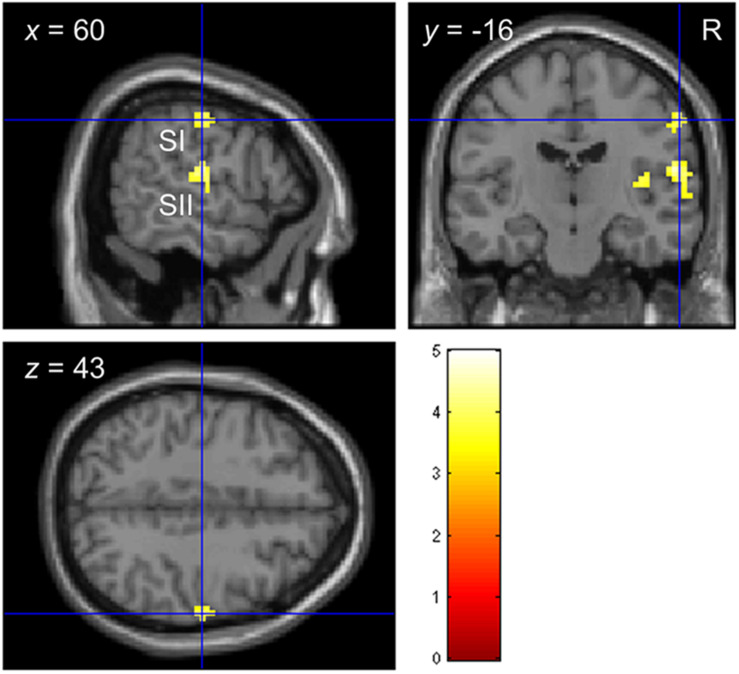
Functional localization of touch-related brain regions in Experiment 2. Clusters in primary and secondary somatosensory cortices (SI and SII, respectively) localized by the contrast *envelope > fixation* with *p* < 0.05 uncorrected at the cluster level.

Consistent with previous studies (Christova et al., 2013; Chung et al., 2013; Kim et al., 2016), stimulating the left index fingertip with the tactile envelope stimulus increased brain activation in contra-lateral (right) primary and secondary somatosensory cortices (SI and SII, respectively), relative to a visual-fixation baseline. Figure 3 shows the clusters in SI and SII, with some evidence that the cluster in SI mapping closely to the somatotopic finger regions in Penfield’s homunculus (Schott, 1993). This tactile stimulation also increased brain activation in ipsi-lateral (left) superior temporal sulcus (STS) and inferior frontal gyrus (IFG) (Hegner et al., 2010, 2017; Nordmark et al., 2012; Wei and Bao, 2013; Kim et al., 2019). Table 4 lists all the clusters from this contrast.

**TABLE 4:** Functional localization of touch-related brain regions in Experiment 2.

				Peak MNI coordinates		
Structure	Cluster size (voxels)	Cluster *p*	*Z*-score	*x*	*y*	*z*
**Envelope > fixation**						
Primary somatosensory cortex (SI)	21	0.043	3.543	60	−16	43
Secondary somatosensory cortex (SII)	84	<0.001*	4.100	45	−22	13
Inferior frontal gyrus (IFG)	42	<0.001*	4.068	45	29	−2
Superior temporal gyrus (STG)	28	0.022	3.858	−60	−40	16
**F200 > fixation**						
STG	200	<0.001*	5.341	54	−16	1
STG	142	<0.001*	4.392	−54	−28	4

*All clusters localized by the contrast envelope > fixation with p < 0.05 uncorrected at the cluster level, and the contrast F200 > fixation with p < 0.05 FDR-corrected at the cluster level. For each cluster, we report the brain structure, the number of voxels in that cluster, its uncorrected p-value, and the Z-score and MNI coordinates for the peak voxel within that cluster. Note that negative x-coordinates refer to the left hemisphere. ^∗^FDR-corrected p-values at the cluster level.*

For the tactile sinusoidal stimuli, only stimulation of the left index fingertip by the F200 stimulus (i.e., carrier frequency = 200 Hz) increased brain activation in left and right superior temporal gyrus (STG) relative to a visual-fixation baseline (see Table 4). Although the tactile stimuli were not audible, previous studies have found that tactile frequencies in the audible range (>∼12 Hz) can activate STG (e.g., Nordmark et al., 2012). We did not find any activation to the F60 and F120 stimuli, however, in this study, we modulated the amplitude with a 2 Hz sinusoidal function which decreased overall power which may have differentially affected brain activation to the three sinusoidal stimulus types.

For each ROI and participant, we extracted the beta estimate from the peak voxel identified from the ROI analysis. We then correlated the beta estimates with the three parameters from the second-order polynomial fit (Eq. 2). As shown in Table 5, there were no significant correlations between brain activation in any of the brain regions and the parameter values (after correction for multiple correlation tests).

**TABLE 5:** Regions of interest correlation between brain activation and detection performance in Experiment 2.

	Quadratic (*p*_1_)	Linear (*p*_2_)	Constant (*p*_3_)
**Envelope > fixation**			
SI	0.33	0.57**	–0.03
SII	–0.09	–0.11	0.44*
STG (left)	0.06	–0.13	0.28
IFG	0.17	–0.38	–0.08
**F200 > fixation**			
STG (left)	0.27	–0.33	–0.30
STG (right)	0.19	–0.32	0.01

*Pair-wise Pearson correlation (r) between participants’ parameters of the second-order polynomial fit (Eq. 2) and beta estimate from the peak voxel in each ROI from the contrasts envelope > fixation and F200 > fixation. There were no significant correlations between brain activation and any of the parameters for all brain regions. ^∗∗^p = 0.01 (uncorrected), ^∗^p = 0.06 (uncorrected), all other ps > 0.11 (uncorrected).*

#### fMRI Whole-Brain Multiple Regression

**FIGURE 4:**
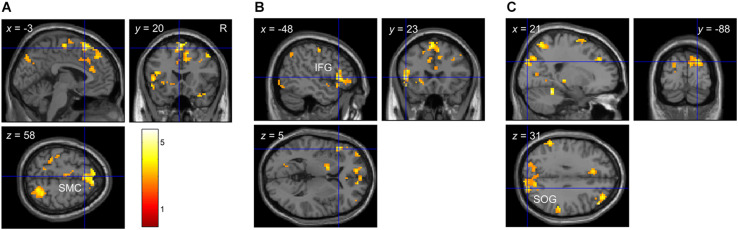
Whole-brain correlation between brain activation and detection performance in Experiment 2. Clusters which showed a significant correlation between brain activation and detection performance with *p* < 0.05 FDR-corrected at the cluster level. **(A)** Cluster in bi-lateral SMC. **(B)** Cluster in left IFG. **(C)** Cluster in right SOG. Note that brain activation was based on the contrast *envelope > fixation*, and performance was based on the parameters from the second-order polynomial fit (Eq. 2) to each participant’s cumulative d’ data in the low tactile-grating intensity. The linear and quadratic trends were largest at this intensity.

At the behavioral level, there were significant linear and quadratic trends in participants’ performance as a function of bar width (i.e., linear and quadratic tactile sensitivity functions). To determine if any brain regions correlated with these behavioral trends, we used 1-sample *t*-tests to localize clusters which significantly correlated with the quadratic (*p*_1_) or linear (*p*_2_) parameters of Eq. 2. Figure 4 and Table 6 show the results from these analyses. There were significant clusters in bi-lateral supplementary motor cortex (SMC), left inferior frontal gyrus (IFG) and right superior occipital gyrus (SOG) whose brain activation correlated with the linear fit parameter. Given the exploratory nature of our preliminary fMRI study, we also report in Table 6 additional clusters that had *p* < 0.05, uncorrected at the cluster level. For the linear parameter, there were additional clusters in motor and parietal cortex, as well as insular and cingulate cortex in the frontal lobe. For the quadratic parameter, there were clusters in the parietal lobe including one that extended into the somatosensory cortex. There were no clusters which correlated with the constant term (*p*_3_).

**TABLE 6:** Whole-brain correlation between brain activation and detection performance in Experiment 2.

				Peak MNI coordinates		
Structure	Cluster size (voxels)	Cluster *p*	*Z*-score	*x*	*y*	*z*
**Linear (p_2_)**						
Supplementary motor cortex (SMC)	306	0.004*	4.126	3	14	58
Inferior frontal gyrus (IFG)	184	0.043*	4.019	−48	23	5
Superior occipital gyrus (SOG)	341	0.004*	3.369	21	−88	31
Anterior cingulate gyrus (CingG)	150	0.002	3.768	6	35	10
Middle frontal gyrus (MFG)	83	0.017	3.709	48	38	31
Motor cortex	141	0.003	3.497	−33	−19	46
Superior parietal lobule (SPL)	119	0.005	3.420	21	−64	61
Frontal pole	73	0.023	3.415	−12	59	−8
Hippocampus	77	0.020	3.342	27	−37	1
Anterior insula (ins)	56	0.043	3.308	−36	11	−5
**Quadratic (p_1_)**						
SI	88	0.014	3.474	30	−25	45
Superior marginal gyrus (SMG)	88	0.014	3.256	57	−37	34

Clusters showing significant correlations with parameters from the second-order polynomial fit (Eq. 2) to participants’ cumulative d’. Note that brain activation was based on the contrast envelope > fixation. Note that negative x-coordinates refer to the left hemisphere. *FDR-corrected p-values at the cluster level.

## Discussion

The three-dimensional micro-structure of physical surfaces produces frictional forces that provide sensory cues about the properties of felt surfaces (Gueorguiev et al., 2017). These properties are important for interacting with objects and making inferences about their material composition. In this study, we used electro-static friction generated on a haptic-feedback touchscreen (Tanvas, Inc.) to simulate three-dimensional gratings with different heights (intensity) and bar widths (spatial frequencies). Blind and sighted observers actively scanned the touchscreen with their dominant index fingertip to detect the gratings by touch alone (Experiment 1). A separate group of sighted but blindfolded observers also participated in a preliminary fMRI study in which we applied different types of vibrotactile stimulation to their left index fingertip, and correlated their brain activation from this stimulation to their detection performance on the grating task outside the scanner (Experiment 2).

At the behavioral level, we found that observers have high sensitivity to detect simulated tactile gratings and, importantly, that they showed strong linear and quadratic tactile sensitivity functions across both experiments. The performance was above chance for all bar widths (minimum cumulative d’ ≈ 1.0, ∼70% accuracy) even at the low tactile-grating intensity used. This intensity represented ∼4% of the touchscreen’s electro-static output range (10 units out of 256 units). Observers were close to the ceiling at both the medium and high tactile-grating intensity used (cumulative d’ > 2.8, >90% accuracy). The medium intensity represents ∼11% of the touchscreen’s output range (30 units out of 256 units). Notably, we found that detection performance showed both linear and quadratic dependency on bar width (when performance was not at the ceiling). We fitted a second-order polynomial function to observers’ sensitivity data to capture this dependency. Based on these fits, the estimated tactile sensitivity peaked at an average bar width of 2.0 mm for blind observers, 2.1 mm for sighted observers and 1.1 mm for blindfolded observers in Experiment 1; and 1.3 mm for blindfolded observers in Experiment 2. Previous studies using haptic-feedback touchscreens (Bau et al., 2010; Vardar et al., 2016, 2017) showed a quadratic dependence of sensitivity on spatial and temporal frequency. It is worth noting that Vardar et al. (2016, 2017) used a variety of waveforms such as sine, saw-tooth and square waveforms, and they tightly controlled finger movement to lateral sweeps at approximately 50 mm/s. Although we only used a square waveform and could not accurately compute finger velocity (due to a coding error), our tactile sensitivity results for blind and sighted participants were similar to these previous studies.

Taken together with recent studies using haptic-feedback technology (Bau et al., 2010; Vardar et al., 2016, 2017), our results are consistent with previous tactile studies using physical stimuli. In the spatial domain, most studies using physical stimuli (e.g., bumps and grooves) showed a linear relationship between tactile sensitivity and spatial properties of physical micro-surfaces such as spatial orientation, gap width and groove size (Johnson and Phillips, 1981; Gibson and Craig, 2002, 2006; Craig et al., 2008). For example, Gibson and Craig used two tasks of tactile spatial acuity—grating-orientation and smooth-grooved discrimination—to show that discrimination accuracy depended on groove width (inversely proportional to spatial frequency), as well hand location (e.g., fingertip vs. palm) and intensity of the stimulus. Interestingly across these studies, there were conditions in which discrimination accuracy also showed a small quadratic relationship with groove width in addition to a strong linear relationship (Gibson and Craig, 2006; Craig et al., 2008). For example, Connor and Johnson (1992) showed a quadratic relationship with texture dot spacing when observers judged texture roughness. In the frequency domain (e.g., physical vibrations at different frequencies), previous studies showed a predominantly quadratic dependence of sensitivity on temporal frequency (Mountcastle et al., 1972; Brisben et al., 1999). Many of these psychophysical studies are in line with neurophysiological studies which show a correlation between perceived roughness and the firing rates of mechanoreceptor afferents (Connor et al., 1990). Thus our results help to extend the perceptual mechanisms for tactile discrimination to simulated tactile stimuli based on electro-static friction.

To the best of our knowledge, this is the first study to compare tactile sensitivity with haptic-feedback technology between blind and sighted observers. We did not find detection-performance differences between the two visual groups, consistent with several previous studies which used physical groves, bumps or textured surfaces (Heller, 1989; Grant et al., 2000; Alary et al., 2009). However, others studies have shown better performance by blind compared to sighted observers (Stevens et al., 1996; Van Boven et al., 2000; Goldreich and Kanics, 2003, 2006). Some of the differences observed between studies may be related to the stimuli and tasks used. It would therefore be interesting in future studies to compare blind and sighted observers’ performance using complex tactile stimuli and tasks, rather than the simple grating detection task used in the present study. We note that there are both age and tactile-acuity (as assessed by the mircro-filaments) differences between our blind and sighted observers (see Table 1). Furthermore, blind participants differed in age of blindness onset (including a small number of congenitally blind participants), cause of blindness and Braille literacy. Thus a limitation in our study is that we have heterogeneous sample populations. However despite these differences, there were no significant effects of visual group on detection performance nor did visual group interact with intensity or bar width (spatial frequency). Overall, our behavioral results in conjunction with previous work suggest that similar perceptual mechanisms may operate for real and simulated touch sensations in blind and sighted observers, leading to the observed linear and quadratic tactile sensitivity functions observed in both visual groups. We further note that across both experiments, we tested a large number of participants (N = 115), a large number of both sexes and a wide age range between 18 and 86. Thus our study provide greater generality despite the heterogeneity of our samples.

We note that there is a relationship between spatial and temporal frequencies (Cascio and Sathian, 2001); this relationship is mediated by the speed with which observers moves their finger when scanning surfaces. However, Boundy-Singer et al. (2017) recently showed that texture perception can be invariant to speed, which takes into account the adaptation profile of mechanoreceptors and their spatial distribution throughout the fingertip. The authors used a variety of natural textures such as sandpaper and fabrics. Importantly, they also used periodic textures such as raised dots at regular spacing that were used in other studies (e.g., Lederman, 1974; Cascio and Sathian, 2001; Yoshioka et al., 2011). This allowed Boundy-Singer et al. to generalize speed invariance results with previous studies to real-world textures. In future studies, we could move the touchscreen (as in Boundy-Singer et al., 2017) or guide finger scan speed during experimental trials (e.g., by visual cues or by a robotic arm, Vardar et al., 2016; Gueorguiev et al., 2017; Vardar et al., 2017). The natural variability of finger movements in our study is closer to real-world movements (i.e., non-guided) which is informative from the perspective of using the tablet as a future visual-aid device (Bateman et al., 2018). Informally, the Supplementary Figures suggest that blind, sighted and blindfolded participants explored the touchscreen in a similar way. That said, future work is needed to investigate how finger movements affect tactile perception of electro-static friction more generally.

In Experiment 2, we conducted a preliminary fMRI study to identify possible brain regions that may be play a role in processing electro-static friction used to simulate the three-dimensional gratings. In the scanner, we stimulated observers’ fingertip with a stationary 120 Hz vibrotactile signal whose amplitude was modulated by a low-frequency (0–30 Hz) envelope to functionally localized brain regions that respond to physical vibrations, particularly SI and SII (e.g., Kim et al., 2016). We also functionally localized left and right STG using a stationary 200 Hz vibratory signal whose amplitude was modulated by a 2 Hz sinusoidal function (e.g., Nordmark et al., 2012). We then correlated brain activation in these ROIs to observers’ tactile sensitivity functions obtained outside the scanner. Contrary to our hypothesis, brain activation in SI and SII did not correlate with either the linear or quadratic parameters from the polynomial fit to observers’ detection performance. Rather, we found that brain activation in regions beyond somatosensory cortex correlated with these parameters. These regions included SMC, IFG, and SOG. The SMC and frontal cortex (e.g., insular or cingulate cortex), along with the intra-parietal sulcus, have been implicated in perceptual decisions about tactile texture patterns, independent of motor responses (Zhang et al., 2005; Hegner et al., 2010, 2015, 2017). Other fMRI studies show that roughness judgments of sandpaper can be decoded from patterns of brain activation in SMC, among other brain regions (Kim et al., 2015); and that active compared to passive explorations of surfaces can increase BOLD responses in frontal and occipital cortices (Simoes-Franklin et al., 2011). Kim et al. (2015) further showed that brain activity in SMC correlated with observers’ performance on an independent task outside the scanner.

Interestingly we found that observers’ detection performance with simulated tactile gratings correlated with brain activation in visual areas in the occipital lobe (SOG). There is evidence that tactile explorations of objects can recruit brain regions subserving vision in sighted observers (occipital tactile-visual area; Amedi et al., 2001; Monaco et al., 2017). Monaco et al. (2017) found stronger activation in retinotopic occipital pole when sighted observers explored objects with their finger in the dark compared to when they visually explored the same objects. Similarly using a roughness discrimination task that did not strictly involve vision, Simoes-Franklin et al. (2011) found increased activation in lingual gyrus when observers actively explored the surface relative to when they passively felt the surface as it was moved for them. Finally, several studies have shown that visual deprivation due to blindness can lead to the recruitment of occipital cortex (Sadato et al., 1996; Cohen et al., 1999; Melzer et al., 2001; Stilla et al., 2008; Siuda-Krzywicka et al., 2016). In our study, we did not scan blind observers but, given our fMRI results, it would be informative to scan this group in future studies. We acknowledge that it is currently not possible to directly measure brain responses to electro-static friction in tactile-related regions using fMRI. Here we used a correlational approach in our preliminary fMRI experiment to address this limitation. In future work, electroencephalography (EEG) is another approach that can be used to measure scalp potentials in response to electro-static friction. For example, there is evidence that somatosensory evoked potentials measured from scalp topography opposite the side of physical touch have their sources in SI (Hari et al., 1990; Zopf et al., 2004; Montoya and Sitges, 2006). Other potential tactile-related brain regions can be estimated by EEG source localization (see Michel and Brunet, 2019, for a review). The trade-off, however, is that this method has much poorer spatial resolution than fMRI. Overall, our behavioral and imaging results suggest that an extended somatosensory brain network may be involved in processing real and simulated touch sensations.

## Conclusion

Consistent with previous behavioral and fMRI studies, our results show (1) that both blind and sighted observers are highly sensitive to simulated tactile gratings based on electro-static friction; (2) that their sensitivity shows a linear and quadratic dependency on spatial frequency; and (3) that touch-related activation in a network of brain regions beyond somatosensory cortex correlated with observers’ detection performance. These findings can be embedded within an initial low-level exploration stage of a hierarchical system for touch (Carbon and Jakesch, 2013; Breitschaft et al., 2019). Future studies can explore later stages of the hierarchy. Our results suggest that digitally manipulating frictional forces to simulate three-dimensional surface micro-structure can allow for the generation of more complex tactile “scenes” to investigate later assessment and evaluation stages of the hierarchy. There are spatial limitations using tactile stimulation by electro-static friction. For instance, Bateman et al. (2018) showed using the Tanvas touchscreen that the accuracy to locate a small 120-pixel target at one out of 30 possible locations was approximately 70% (although this was still well above chance). Similarly, the current version of the Tanvas touchscreen would not have the spatial resolution to simulate Braille letters. That said given that similar perceptual and neural mechanisms may be recruited for real (e.g., vibrations) and simulated (e.g., digitally manipulated frictional forces) touch sensations, such haptic-feedback technology can be used in the future to enhance the multi-media experience for those with and without visual impairments (Bateman et al., 2018).

## Data Availability Statement

The raw data supporting the conclusions of this article will be made available by the authors, without undue reservation, to any qualified researcher.

## Ethics Statement

The studies involving human participants were reviewed and approved by the Newcastle University and Helwan University Ethical Reviews Committee. The patients/participants provided their written informed consent to participate in this study.

## Author Contributions

WA-A, PD, and QV contributed to the design, analyses of the data, and drafting the manuscript. AS, CB, and MN collected the detection performance data. JS collected the fMRI data. AA programmed the experiments and contributed to data processing. All authors commented on drafts.

## Conflict of Interest

The authors declare that the research was conducted in the absence of any commercial or financial relationships that could be construed as a potential conflict of interest.
