# Supplementary material for: Detection of Simulated Tactile Gratings by Electro-Static Friction Show a Dependency on Bar Width for Blind and Sighted Observers, and Preliminary Neural Correlates in Sighted Observers
Source: Front Neurosci. 2020 Oct 14;14:548030. doi: 10.3389/fnins.2020.548030 (PMC7591789; doi:10.3389/fnins.2020.548030)

Supplementary data for "Detection of simulated tactile gratings by electro-static friction show a dependency on bar width for blind and sighted observers, and preliminary neural correlates in sighted observers"

Positional heat maps by visual groups in Experiments 1 and 2

Finger positions (*x*- and *y*-coordinates) were sampled every 40 ms. We were able to accurately record finger position but, due to a coding error, we were not able to accurately compute finger velocity because we did not record periods when participants lifted their scanning finger off the touchscreen. For descriptive purposes, we therefore could only present positional heat maps of the scanning finger for the different visual groups in Experiments 1 and 2. Each plot (2048 pixels x 1536 pixels) represents mean "duration" the pixels were in participants' finger scan path as a function of intensity and bar width. The mean "duration" was computed by (1) convolving raw *x*- and *y*-coordinates with a 2D Gaussian function (sigma = 50 pixels) to smooth the sparse image for each trial, (2) summing the smoothed images across all participants and trials, and (3) dividing the summed image by the total number of trials. Warm colours represent longer durations than cool colours.

Experiment 1

Blind (N = 46)

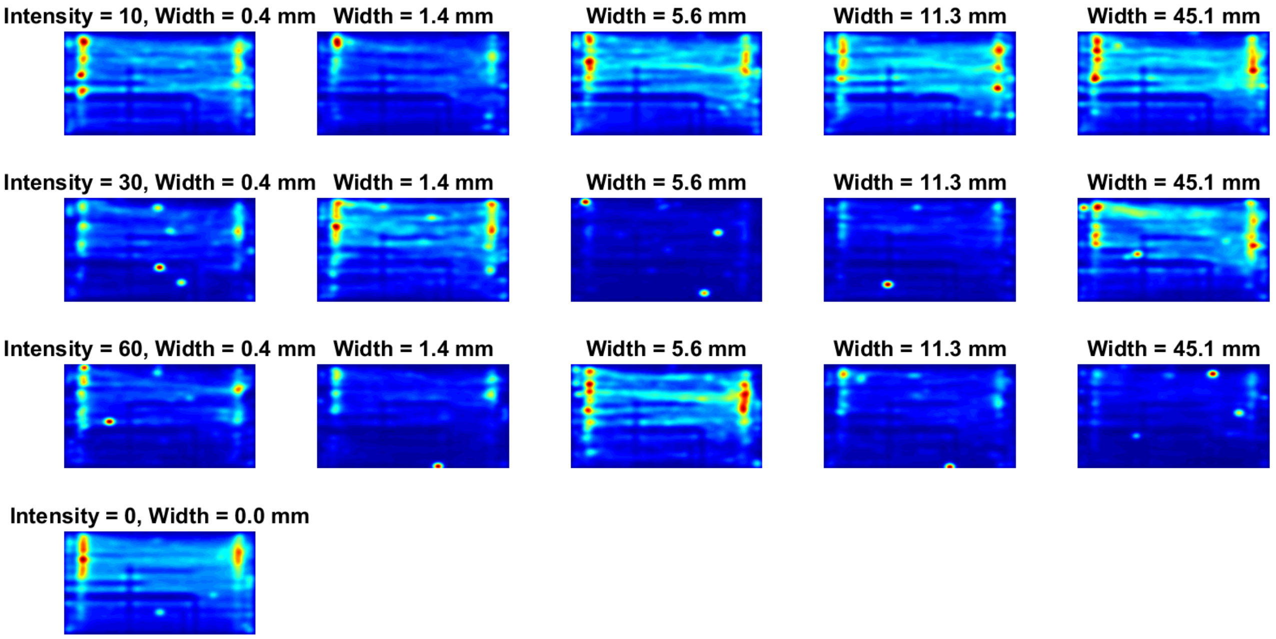

Sighted (N = 46)

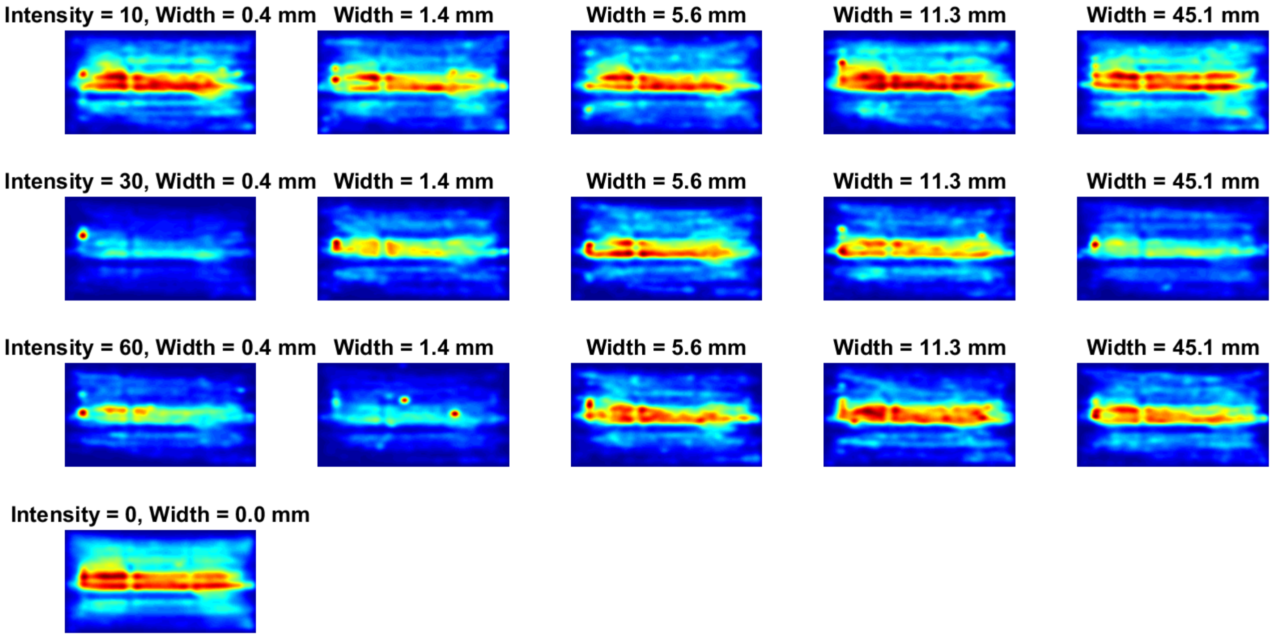

Blindfolded (control, N = 22)

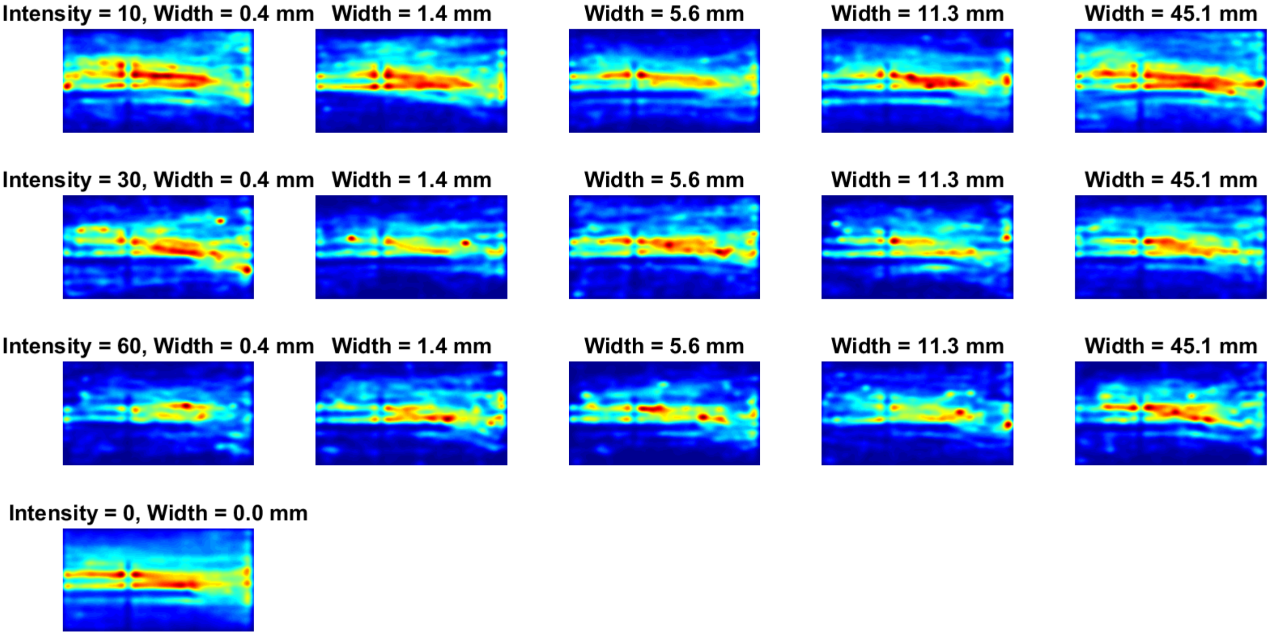

Experiment 2

Blindfolded (N = 19)

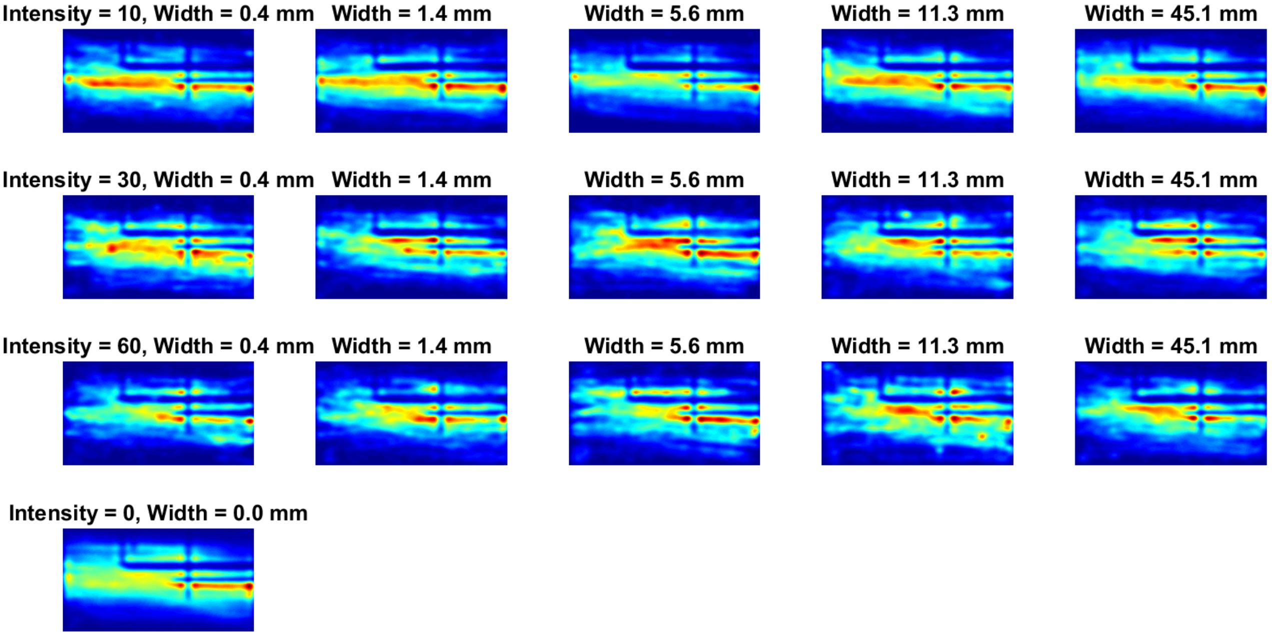

All groups across Experiments 1 and 2

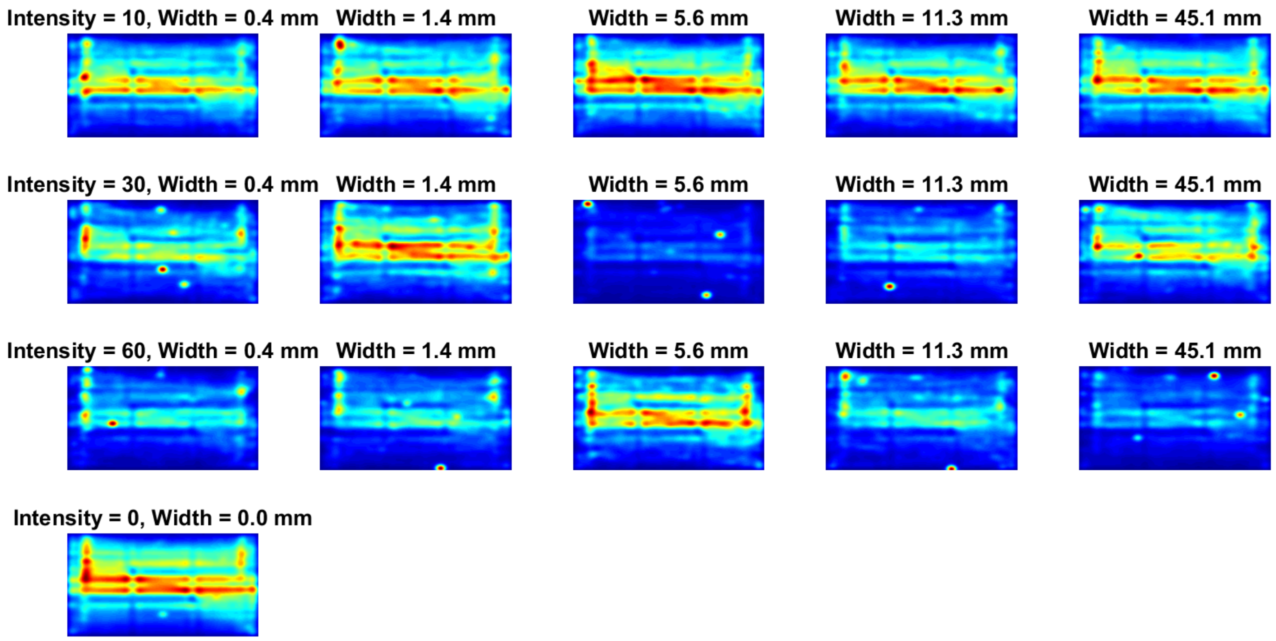

Supplement: Supplementary file 1 [file Data_Sheet_1.PDF]
